# Orchestral role of lipid metabolic reprogramming in T-cell malignancy

**DOI:** 10.3389/fonc.2023.1122789

**Published:** 2023-05-15

**Authors:** Arundhati Mehta, Yashwant Kumar Ratre, Vivek Kumar Soni, Dhananjay Shukla, Subhash C. Sonkar, Ajay Kumar, Naveen Kumar Vishvakarma

**Affiliations:** ^1^ Department of Biotechnology, Guru Ghasidas Vishwavidyalaya, Bilaspur, Chhattisgarh, India; ^2^ Trivitron Health Care Pvt. Ltd., Visakhapatnam, India; ^3^ Multidisciplinary Research Unit, Maulana Azad Medical College, University of Delhi, New Delhi, India; ^4^ Department of Zoology, Banaras Hindu University, Varanasi, India

**Keywords:** T cell malignancies, lipid metabolism, fatty acids, lipid droplets, lipid rafts

## Abstract

The immune function of normal T cells partially depends on the maneuvering of lipid metabolism through various stages and subsets. Interestingly, T-cell malignancies also reprogram their lipid metabolism to fulfill bioenergetic demand for rapid division. The rewiring of lipid metabolism in T-cell malignancies not only provides survival benefits but also contributes to their stemness, invasion, metastasis, and angiogenesis. Owing to distinctive lipid metabolic programming in T-cell cancer, quantitative, qualitative, and spatial enrichment of specific lipid molecules occur. The formation of lipid rafts rich in cholesterol confers physical strength and sustains survival signals. The accumulation of lipids through *de novo* synthesis and uptake of free lipids contribute to the bioenergetic reserve required for robust demand during migration and metastasis. Lipid storage in cells leads to the formation of specialized structures known as lipid droplets. The inimitable changes in fatty acid synthesis (FAS) and fatty acid oxidation (FAO) are in dynamic balance in T-cell malignancies. FAO fuels the molecular pumps causing chemoresistance, while FAS offers structural and signaling lipids for rapid division. Lipid metabolism in T-cell cancer provides molecules having immunosuppressive abilities. Moreover, the distinctive composition of membrane lipids has implications for immune evasion by malignant cells of T-cell origin. Lipid droplets and lipid rafts are contributors to maintaining hallmarks of cancer in malignancies of T cells. In preclinical settings, molecular targeting of lipid metabolism in T-cell cancer potentiates the antitumor immunity and chemotherapeutic response. Thus, the direct and adjunct benefit of lipid metabolic targeting is expected to improve the clinical management of T-cell malignancies.

## Introduction

Lipid makes essential components in cells, providing structural moieties and energy reserve. Apart from these, lipids and their derivatives serve as signaling molecules and regulate the functional and behavioral phenotypes of cells (1). The alterations in lipid metabolism serve as instrumental gear in the onset of malignancies and in maintaining the “hallmarks of cancer” (1, 2). The rewired lipid metabolism has been reported in various cancer forms including those of hematological malignancies (3). Among hematological malignancies, the cancer of T cells has the uniqueness of being derived from the prime cell in the immune system. The metabolic intricacy especially in lipid metabolism plays an underlying role in maintaining the phenotypic characteristics in subsets of T cells (4, 5). The inter-regulated pattern of lipid metabolism is also involved in directing the amplitude, magnitude, and temporality of T-cell response (6). Accumulation, storage, and oxidation arms of lipid metabolism are differentially operated during phases of functional and phenotypic activations of T cells (7).

Lipid metabolic reprogramming plays an instrumental role in the transformation and maintenance of T-cell malignancies (8, 9). The various fragments of lipid metabolism are modulated in cancer cells derived from T lymphocytes (10, 11). The high membrane requirement of rapidly dividing cells demands *de novo* biosynthesis of fatty acids and other lipid molecules. The synthesis of fatty acids by the activity of fatty acid synthase (FASN) is upregulated in malignant cells of T-cell origin (12). The elevated expression and activity of FASN in T-cell malignancies are linked with many other hallmarks of cancer (13). The uptake through fatty acid translocase (FAT), also known as CD36, also contributes to the cellular pool of lipids in various types of cancers (14). The sequestration of freely available lipid molecules serves as compensating means during insufficiency or pharmacological inhibition of FASN (15, 16). The uptaken lipid molecules or fatty acids generated through the activity of FASN are utilized to generate structural components or bioactive molecules. The excess lipid content is stored in lipid droplets (also known as adiposomes), initiated in the membrane of the endoplasmic reticulum (17). These droplets serve as lipid reserves required during robust energy response. Moreover, lipid droplet abundance is linked with cancer cell aggressive behavior even in malignancies of T cells (18). The upregulated biosynthesis of cholesterol and other lipids like sphingolipids in T-cell cancer have an advantageous impact through modulating membrane strength, dynamics, and spatiometric composition (19, 20).

The oxidation of lipids provides small biosynthetic precursors. Acetyl CoA is one of the major molecular entities generated after the complete oxidation of long-chain fatty acids (21, 22). Moreover, the acetyl CoA generated through fatty acid oxidation (FAO) links other metabolic pathways by fueling the tricarboxylic acid (TCA) cycle (22, 23).

The spatiometric abundance of lipid moieties holds the surface expression of protein favoring cell survival in hematological malignancies (24, 25). The benefits of restructured metabolism of lipids confer resistance to the action of chemotherapeutic drugs (26) and antitumor immunity and aid in cancer cell evasion from immune-mediated destruction (11, 27). The indispensability of lipid metabolism in the cellular physiology of T-cell cancer makes them targetable for therapeutic intervention. Various approaches have shown promising success both in preclinical and clinical settings (28, 29). Lipogenic, as well as oxidative metabolism of lipids, has been targeted in T-cell cancer (22, 30). As the metabolic pathways are intervened and compensate for each other, combinatorial and dual targeting has provided enhanced success (31). Understanding various dimensions of lipid metabolism in the life of T cells, their subsets, and their rewiring in malignant T cells is expected to open avenues for therapeutic targeting of molecular players within.

## Metabolism in T cells: the lipid perspective

T cells drive acquired immunity against infections and other immunological threats, but their outage can also play a role in the development of cancer and autoimmune diseases. T-cell activity and lineage decision are influenced by metabolic adaptability in response to the immune system and microenvironmental stimuli (32). There is a shift in the metabolic pattern of T cells to meet the dynamic requirements at different phases of development, activation, clonal expansion, and memory acquisition that significantly differs in various functional subsets of T cells (33). Engagement of T-cell receptors with antigens in supramolecular activation complex (SMAC) triggers an energy- and biosynthesis precursor-demanding process of blastogenesis (growth in cell size) and subsequent robust cell division (34). To fulfill these demands, antigen-encountered T cells reconfigure their metabolism and preferably use aerobic glycolysis as a major source of energy (ATP synthesis) (6).

In the field of immune metabolism, a lingering question that remains unanswered is whether T-cell differentiation is promoted by ambient metabolic resource accessibility or whether the metabolic requirements are set by intrinsic cellular systems, determined by the environment (6). However, experimental evidence suggests some lines of bilateral regulation (6, 33).

## T-cell activation and lipid metabolism

During the proliferation of T cells, the intermediatory metabolites generated from pathways aligned with glycolysis (PPP and TCA cycle) include ribose-5-phosphate and citrate (35). These serve as forerunning precursors for the synthesis of biological macromolecules, the formation of membranes, and organellar biogenesis (6, 36). In addition to components of SMAC, microenvironmental cues as a function of cytokines are major determinants of functional activations of T cells (37). Nevertheless, T-cell fate is substantially influenced by access to metabolic and nutrient resources (33, 38).

During the naïve and resting phase of T cells, oxidative phosphorylation (OXPHOS) and FAO are major bioenergetic sources. Such cellular phenotype metabolically shifts during activation signal engagement in SMAC leading to the proliferation of T cells (35, 39). PI3K and mTOR activation are major signaling mediators of T-cell activation. mTOR serves as a junctional point for the proliferation of cells as well as for modulating metabolic outline. Through activation of c-myc and HIF-1α, the T-cell activation signals upregulate the expression of glucose transporters and enzymes of glycolysis (40, 41). This ensures the accelerated aerobic glycolysis in T cells while transitioning from naïve to effector cell phenotype. Aerobic glycolysis in cancer cells, known as the “Warburg effect”, is also considered a metabolic hallmark (42, 43). Aerobic glycolysis is the product of genetic changes in cancer cells leading to dysregulated metabolic programming (43). However, aerobic glycolysis in T cells is attributed to a concerted regulation of cellular physiology. Metabolome analysis of activated T cells endorses that the biosynthesis of fatty acids also goes along with biosynthetic pathways of amino acids and nucleic acids (42). Nevertheless, augmented fatty acid (FA) biosynthesis and FAO downregulation indicate the fundamental role of lipid metabolism in T-cell activation and function (44). mTOR-mediated stimulation of transcription factor sterol regulatory element-binding proteins (SREBPs) upregulates the expression of enzymes for FA biosynthesis (45, 46). FASN is one of the major enzymes under the regulation of transcription factor SREBPs. Among many others, acetyl-CoA carboxylase (ACC1), and hydroxy-methylglutaryl-CoA reductase (HMGCR) are key enzymes in FA synthesis. SREBP-driven cholesterol production is the gateway for T-cell blastogenesis during functional activation (47). Liver X receptor (LXR), a cholesterol regulatory element, is also demonstrated to be vital in activation-induced T-cell proliferation (48).

## Lipid metabolism in T-cell subsets

T-cell subsets have distinct lipid metabolic operations during their functional phases. Through various specific inhibitions of lipid metabolic steps, the differences in naïve, resting, and functional stages were demonstrated. The inhibition of FA synthesis through knockout or inhibition of specific enzymes was not found to largely affect the naïve and resting T cells and their ability to differentiate (49, 50). However, a significant decline in CD8 T cells was observed in the ACC1 deletion experiment (49). Apart from *de novo* synthesis, CD4 T cells sequester exogenous FA through the upregulation of their transporters (51). The exogenously available FA are suggested to upregulate their transporters (including CD36) and mediators in storage (such FA binding proteins (FABPs)) through activation of peroxisome proliferator-activated receptor-gamma (PPARγ) (14). Among many others, GPR43 and GLP84 are suggested as additional promising receptors of FA in CD4 cells (52) However, FA synthesis obviates the dominance of aerobic glycolysis in metabolic reprogramming in T cells during activation (5, 35, 52). The exclusive necessity of FA synthesis for CD8 T cells at the same time as collateral supplementation through the uptake of FA in CD4 T cells indicates the cell type-specific differential role of lipid metabolism (35).

Among the various subsets of CD4 T cells, Th1 and Th2 are predominant. Th17 and Treg cells qualitatively and quantitatively regulate helper T cells as well as other immune cells’ responses (53). The variance in immunometabolism of lipids in these subsets of T cells has been recently reported (6). Most of the helper T-cell subsets (Th1, Th2, and Th17) operate FA synthesis/sequestration and largely depend on aerobic glycolysis for their energy demand (54), while Treg cells have a preference for FA oxidation (6, 53). Th1 cells have the keen requisite for the type and availability of exogenous FA as compared to Th2 cells (55). The prevalence of long-chain FA (LCFA) can promote Th1 subset functions, while polyunsaturated FA (PUFA) can inhibit the production of this subset-specific cytokine production (56). As the differentiation and function of Th1 and Th2 subsets are inter-regulated, the role of lipid metabolism can be sought to have a central role in the fate of the immune response. It will be noteworthy to mention that this subset phenotype activation is not irreversible and have a distinct level of plasticity. Among Th cell subsets, Th17 most profoundly operates *de novo* biosynthesis of FA (57). The inflammatory upregulation through IL1 and IL23 upregulates the FA synthetic machinery in Th17 (58). A decrease in Th17 cell proportion through pharmacological inhibition of FA synthesis indicates the fate-determining role of lipid metabolism (57), nevertheless indicating the role of the availability and metabolism of lipids in plasticity among T-cell subsets. Depletion of ACC1 in T cells favors the upregulation of Foxp3, a Treg-specific transcription factor, even in Th17 differentiating conditions (10). Moreover, PPAR ligands have also been shown to modulate the induction of Treg cells (10). Unlike other T cells, the Treg cells have the functioning of OXPHOS and FAO (59). The cytosolic FA is channelized to FAO in mitochondria by carnitine palmitoyl transferase (CPT) 1A (CPT1A) (60). The suppressive function of Treg cells is fueled by FAO, the generation of anaplerotic moieties for the TCA cycle, and the subsequent driving of OXPHOS (10).

## Memory T cell and lipid metabolism

After the effector phase of cell life, T cells follow the course of memory cells. During the transition of effector cells to memory cells, functional switches are for the second time put into action. However, the second transition is reversed in the manner (shading of effector function). The hyperglycolytic phenotype of effector T cells is halted, and OXPHOS takes over T memory (Tm) cells (35). The lipid metabolism shifts from the synthesis of FA to their oxidation. Inhibition of glycolysis and/or FA synthesis in activated T cells promotes the formation of Tm cells, while the inhibition of these pathways before the activation signal for T cells prevents their functional differentiation. The switching off of the mTOR signal and activation of AMPK-mediated signaling mediate this transition of cell phases along with metabolic shift (61, 62). The abundant mitochondrial activity required to meet FAO and OXPHOS leads to an increase in mitochondrial biomass in Tm cells. This provides a survival/persistence advantage to the Tm cells (35). ACC1 inhibition in activated T cells also favors the formation of Tm cells (63). ACC1 is also involved in the fate-determining step during the differentiation of T cells in subsets after the antigenic encounter (64). It indicates that among many lipid metabolism enzymes, transporters, and regulators, ACC1 has a varied role during different phases of T-cell life (63, 64).

Metabolic events and key molecular players involved in phenotypic activation and effector functions of various subsets of T cells are listed in **Table 1**. Lipid metabolism has an indispensable role in stimulation, activation, differentiation, function, and memory formation in T cells (38, 42, 54, 63).

**Table 1:** Lipid metabolism in functional subsets of T cells.

T-cell subset		Major metabolic events	Key molecular players
**Naïve T cell**		Majorly rely on OXPHOS and FAO	Enzymes of OXPHOS and FAO
**Ag-stimulated T cells**		Aerobic glycolysis, FA synthesis, uptake, and accumulation	mTOR, PI3K, c-myc, HIF, GLUTs, FASN, SREBP, and PPARγ
**CD8^+^ cytotoxic T cells**		Mainly rely on FA and lipid synthesis	FASN, ACC1, HMGCR, and SREBP
**CD4^+^ helper T cells**		FA and lipid synthesis and FA uptake	FASN, SREBPs, CD36, FABPs, GPR43, GLP84, and LXR
	**Th1**	Relatively high FA uptake dominates over FA synthesis	CD36, FABPs, FASN, and LCFA favor Th1 activation
	**Th2**	Relatively low FA uptake, however, dominates over FA synthesis	CD36, FABPs, FASN, and PUFA favor Th2 activation
	**Th17**	Profound FA synthesis and aerobic glycolysis	FASN, ACC1, PDHK, LXR, and 2-HG favor Th17 activation
**Regulatory T cells (Treg)**		Exogenous FA uptake dominates over FA synthesis, OXPHOS, and FAO	Foxp3, FABP5, CPT1, downregulation of ACC1, SREBPs, and SCAP
**Memory T (Tm) cells**		OXPHOS and FAO, FA uptake, and downregulation of FA synthesis	Enzymes of OXPHOS and FAO, FABP4/5, AMPK, and downregulation of ACC1

OXPHOS, oxidative phosphorylation; FAO, fatty acid oxidation; FA, fatty acid.

## Lipid metabolism in T-cell malignancy

Malignancies of T cells have substantial rewiring of metabolism spanning to glycolysis, glutamine addiction, and reprogrammed lipid metabolism (65–67). The unique alteration in lipid metabolism ranging from their *de novo* biosynthesis, uptake of free lipid moieties, and accumulation of lipids in specialized structures (lipid droplets) is commonly observed in paths altered in cancer cells (17, 68). The alterations in catabolic pathways are also observed in malignancies (16). These alterations in lipid metabolic pathways act in a concerted fashion and aid in the phenotypic characteristics of malignant cells (17, 68). Changes in lipid metabolic setup in cancer cells are generally sought as uncontrolled; however, these modulations are well structured through an array of molecular regulators (35, 69). Strenuous adjustments in lipid metabolism have numerous gains in accelerated survival, modulation of survival and death signaling, and resistance to chemotherapy and immunotherapy (26, 70, 71). The contribution of qualitative and quantitative changes in cellular lipid content especially those of membrane aid in the progression of a variety of cancers.

## Cancer lipid metabolism

**Figure 1:**
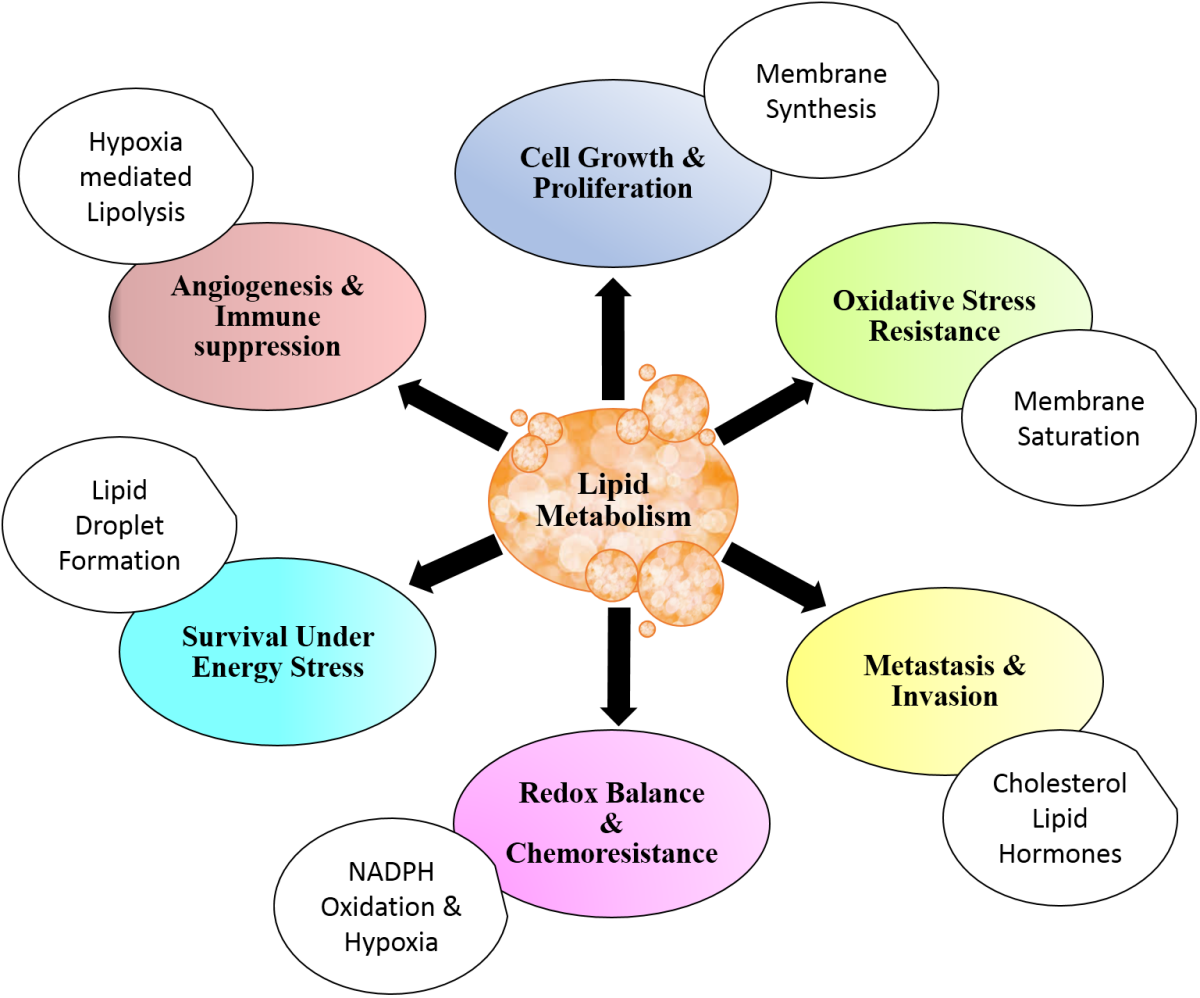
Lipid metabolism and cancer. Lipid metabolism contributes to cancer cell physiology and aggressiveness through modulation of cell survival and death. Altered lipid metabolism also affects angiogenesis and metastasis. Distinctive metabolic setup of lipid aids in resistance to chemotherapy and antitumor immune response. All these benefits are in addition to energy storage and structural contribution for rapidly dividing cells.

For rapidly dividing cells, metabolic alterations are essential to be acquired to meet the prerequisite for cell growth and cell division. The alterations in metabolism are also common in pathways that involve lipids (6, 42, 43). Together with other biological macromolecules, lipids contribute to the structural as well as energetics and cellular signaling of cells undergoing proliferative phases (32). Metabolic alterations are tightly regulated physiological events in normal cells, while they are brought about by dysregulated setup owing to genetic variations in cancer cells (72–75). Several studies demonstrated that malignant cells harness lipid metabolism to support their rapid proliferation and uphold invasion and metastasis (3, 4, 65). The benefits of lipid metabolism in cancer are depicted in **Figure 1**.

The upregulation of *de novo* synthesis of FA in cancer cells is reported by many experimental investigations (76, 77). Targeting the enzymes of FA synthesis has an inhibitory effect on cancer cells, indicating their critical role in cancer cell survival (77). The survival signaling through PI3K/AKT axis favors the upregulated expression of enzymes involved in FA synthesis (1). Moreover, the activation of ATP-citrate lyase (ACLY) is also endorsed by PI3K/AKT signaling (78). The ACLY is the enzyme responsible for the lysis of citrate and the production of Acetyl CoA, the two-carbon precursor for FA synthesis (1, 78). The pool of cellular Acetyl CoA is acetate generated from glucose, glutamine, and other carbon sources (79). The cytosolic acetate is ligated with CoA by acetyl-CoA synthetase (77, 80). Subsequently, the conversion of acetyl CoA into malonyl CoA is favored by the activity of ACC1/2. The transcriptional upregulation of ACC1/2, ACSS, and ACLY is carried out by SREBPs (81–83). Many cancers have SREBP upregulation favoring the lipogenesis enzymes expression in malignant cells (83). The upregulation of ACC1/2, ACSS, and ACLY has been observed in many cancer types (84) Interestingly, ACLY is also involved in nuclear dynamics by providing acetyl CoA for histone acetylation after their nuclear translocation (85). The FASN catalyzes the condensation of small precursor moieties into 16-carbon long fatty acid palmitate (86, 87). The fatty acid molecules synthesized by FASN activity provide a significant fraction of cellular lipid content (87). The FA further undergoes conversions including elongation and desaturation. The palmitate serves as the precursor for cellular non-essential FA content and is converted by FA desaturases (FADS), stearoyl-CoA desaturases (SCD), and FA elongation (65, 88, 89). SUMOylation of FASN can prevent its degradation (88). Moreover, the upregulation of HDAC3 maintains the deacetylated form of FASN in hepatic cancer cells (34, 72, 87). Otherwise, acetylation of FASN by KAT8 (lysine acetyl transferase 8) makes it susceptible to ubiquitin ligation and degradation (90). Proteasomal degradation of FASN is also prevented by a mutation in ubiquitin ligase speckle-type POZ protein in prostate cancer cells (87, 91).

Consecutively, the condensation of acetyl CoA generates 3-hydroxy-3-methylglutaryl–CoA (HMG-CoA) (92). The enzyme HMGCR catalyzes the conversion of HMG-CoA into mevalonate, a rate-limiting step in cholesterol synthesis (93). The upregulated expression of HMGCR is observed in several cancer cell types. The transcriptional regulator of many lipogenic enzymes, SREBP, also elevates the expression of HMGCR (92, 93). The subsequent production of isoprenoid farnesyl pyrophosphate (FPP) serves as a precursor for cholesterol. The squalene generated from FPP is then converted into cholesterol by the enzyme squalene monooxygenase (SM) (94). This enzyme is also under the transcriptional regulation of SREBP and is sought to be a metabolic target for the therapy of cancer (46). The inhibition of either HMGCR or SM has shown promise in anticancer therapy by restrictive cholesterol synthesis (19). The inhibition of HMGCR was also reported to adjunct the activity of immune-checkpoint inhibition therapy by anti-PD1 antibodies (43). These pieces of evidence collectively indicate the crucial role of cholesterol synthesis in not only providing resources for cell growth and division but also aiding in escape from cell death and antitumor immune response.

Various mechanisms are reported to be upregulated for lipid uptake in cancer cells (87). Majorly, the uptake of free FA is carried out by CD36 (fatty acid translocase) or fatty acid transport proteins (FATPs) (45, 87). Low-density lipoprotein (LDL) known as LDL receptor is found to be upregulated in various cancer types and assist in lipid uptake through endocytosis (21). Moreover, the upregulation of FABPs also favors the uptake of FA and their transport (87) and has been reported to have high expression in different cancer cells (50, 87). The balance of FA synthesis and uptake is keenly regulated, as it modulates the relative abundance of saturated and unsaturated FA. The degree of saturation and their relative abundance in turn alter the susceptibility toward reactive oxygen species (ROS)-mediated peroxidation of lipids. FAs are converted to triglycerides and then stored in the form of lipid droplets (LDs) (4). The bilateral traffic of lipids in and out of an LD is dependent on the abundance of lipids, availability of oxygen, and activity of enzymes including DGAT and PLIN (95). Many of these lipid droplets are found upregulated in cancer cells (3).

**Figure 2:**
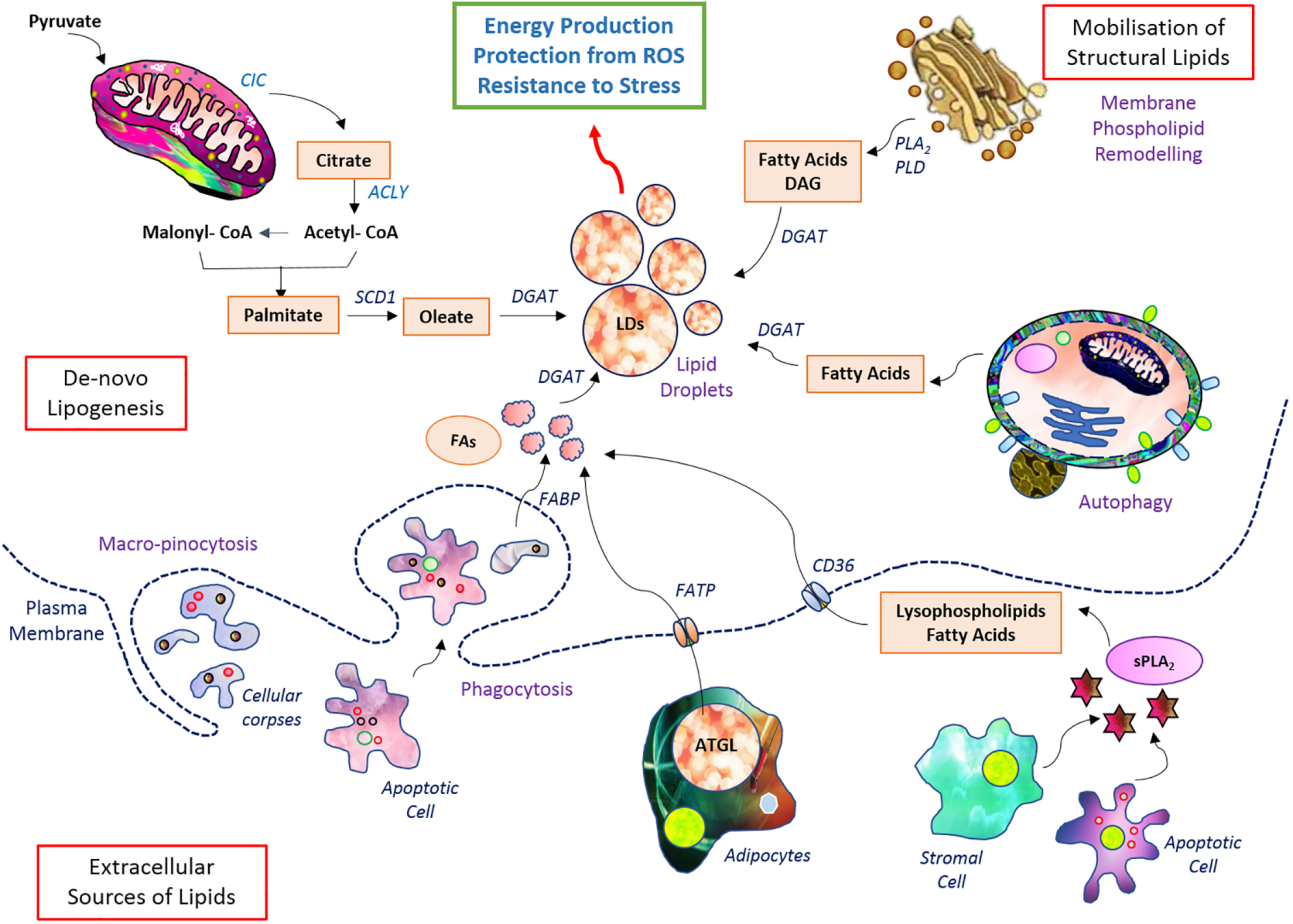
Setup of lipid metabolism in malignant cells. Lipid metabolic pathways have diverse wings of *de novo* biosynthesis, elevated uptake, and storage in lipid droplets. The stored lipid serves as energy reserve during robust demand as well as confers protection from induction of cell death.

Apart from building blocks, accumulated lipids provide precursors for signaling and regulator molecules (96) through the activity of various lipases (phospholipases for phospholipids of membrane) (84, 96) Arachidonic acids, lysophosphatidic acid, and diacylglycerol are a few among many such bioactive molecules generated from the breakdown of lipids and regulate cellular fate (97). Many of these activate the PI3K and RAS signaling axes and favor neoplastic transformations (98). They also favor cancer cell survival, metastasis, drug resistance, and stemness of cancer cells (3, 32). The transport of FA from the cytosol to the inner core of mitochondria is accomplished by CPT1 and CPT2, respectively found in the outer and inner membranes (99) (100). The total lipid pool of cancer cells is contributed by *de novo* biosynthesis and uptake of lipids from cellular exteriors and is stored mainly in the form of lipid droplets (**Figure 2**). Lipid metabolism is integrated with the metabolism of other nutrient sources not only through sharing but also through replenishing intermediates conditionally (101).

## Alterations in lipid metabolism in T-cell cancer

**Figure 3:**
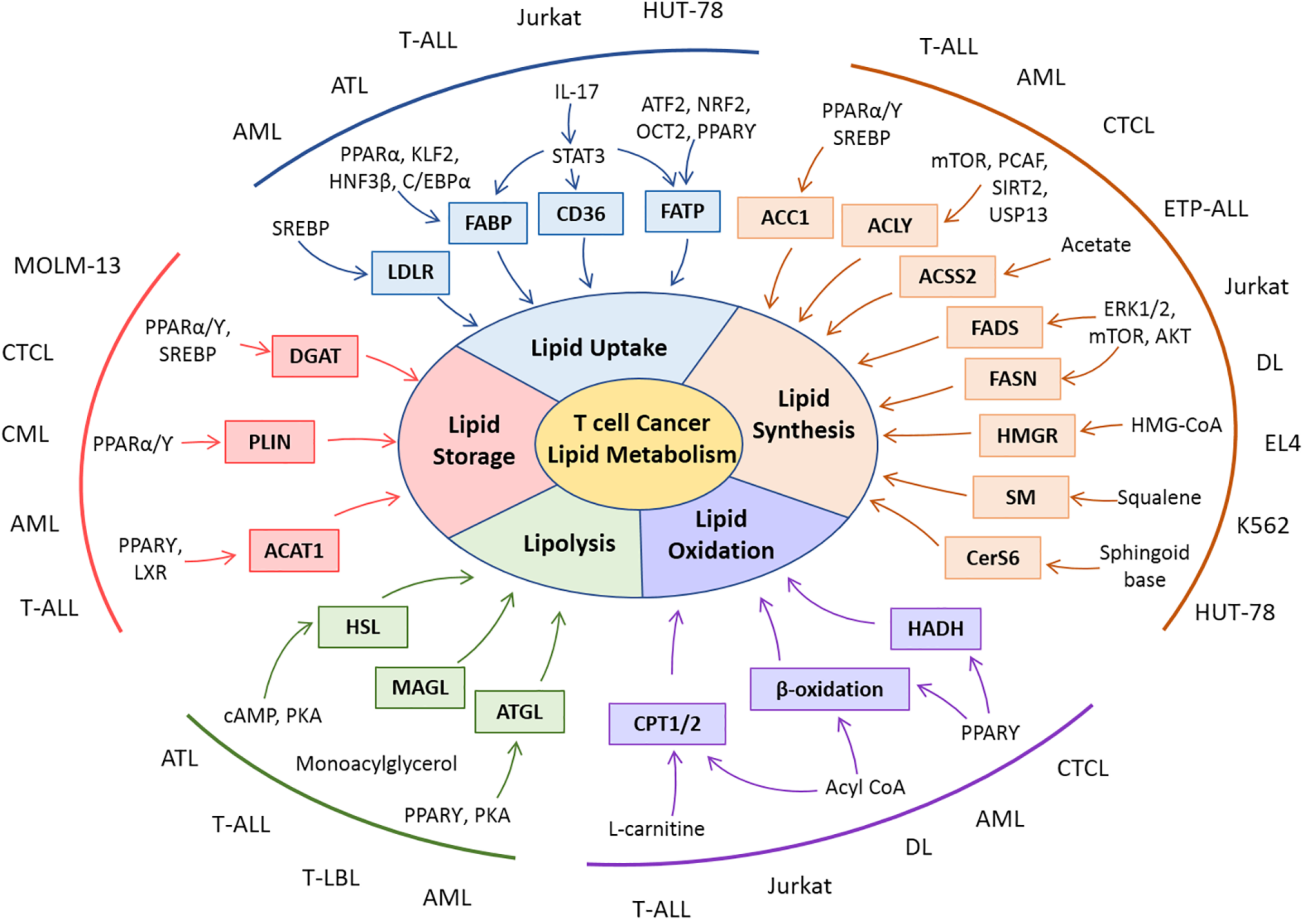
Snapshot of lipid metabolic alterations and their molecular players in T-cell malignancies. Rewiring in various dimensions of lipid metabolisms is reported in clinical and preclinical studies with T-cell malignancies. Patient-derived cells (AML, ATL, CML, CTL, ETP-ALL, and T-LBL) or cell lines (Jurkat, EL-4, DL, HUT-78, K562, and MOLM-13) of T-cell cancers exhibit modulation in lipid metabolic players as well as their regulators.

The modulation of lipid metabolism in T cell-derived malignancies extends to the biosynthesis of lipids through the upregulation of FASN and cholesterol-producing machinery. The upregulation of transcriptional regulators, their mechanistic role, and connections with other metabolic arms favor T-cell cancer in their progression. Accumulation and storage of lipids in droplets and subsequent oxidation of lipids in T-cell cancer have been shown to aid in the progression of cancer cells. Dynamic and intervened connection of lipid metabolism with other metabolic pathways and tumor cell survival is linked in hematological malignancies. Alterations in components of lipid metabolism in T-cell malignancies are illustrated in **Figure 3**.

## Lipid biosynthesis in T-cell cancer

Malignancies of T-cell origin, too, have a high demand for biosynthetic material, and hence, heightened synthetic machinery for precursor molecules is upregulated. Few key enzymes catalyzing the steps of biosynthetic pathways for lipids are found to be upregulated in lymphoma cells including those of T-cell origin. The high cell proliferative ability of lymphoma cells correlates with upregulated expression of FASN (16, 102). Upregulation of FASN in extra-nodal nasal NK/T-cell lymphoma cells was found to correlate with their survival adaptation (103). The upregulation of FASN in T-cell acute lymphoblastic leukemia (T-ALL) patient-derived cancer cells correlates with poor prognosis and drug susceptibility (104). With a murine model of T-cell lymphoma, FASN has been demonstrated to be a targetable enzyme that weakens the chemoresistant amplitude (13, 105). The expression of ACSS2 was also suggested to support the survival of T-cell lymphoma by maintaining the osmotic tolerance of transformed cells (106). Moreover, the SREBP1 level in transplantable T-cell lymphoma was suggested to provide an advantage in cell survival (106). The inhibition of SREBP in cutaneous T-cell lymphoma (CTCL) reduced the FASN expression partially (31). It suggests that FASN has an additional regulatory arrangement, at least in T-cell malignancies.

In anaplastic large-cell lymphoma, nucleophosmin-anaplastic lymphoma kinase phosphorylates the ACLY (107). The phosphorylation statuses of Y682 tyrosine residue of ACLY serve as a control switch for the synthesis of lipid and FAO. Moreover, the ACLY activity was also demonstrated to adjust oncogenesis in anaplastic large-cell lymphoma (107).

The cholesterol synthesis pathway is also found to be modulated in many lymphoma cell types. The proliferative capacity of T-cell lymphoma cells correlates with their HMGR activity and potential to synthesize cholesterol and its esterification (108). Interestingly, the accumulation of intermediary metabolite of the cholesterol synthesis pathway, i.e., squalene, was reported in Jurkat cells (109). Moreover, oncogenic stimulus mediated by wnt signaling triggers the generation of T-cell lymphoma through the upregulation of cholesterol synthesis (110). The syntheses of fatty acid and lipid molecules in cells of T-cell cancer not only offer superior survival and proliferative ability but also play a critical role in oncogenic transformation (**Figure 3**).

## Lipid sequestration in T-cell malignancies

Uptake of lipid moieties from exterior their exterior is shown to affect the cancer cell physiology and assist in upholding stemness in a variety of malignancies. Moreover, the import of FA into cytosol has a critical impact on the malignant transformation of cells (87, 111). Research investigations have shown that lipid uptake is upregulated in lymphoma cells (96). However, most of these research investigations focused to investigate lipid uptake utilizing cancer cells or patient-derived samples of B-cell origin (102). Increased expression of FAT on lymphoma cells is indicated to be a good prognostic marker (16). LDLR also correlates with adverse outcomes in leukemia cells (112).

**Figure 4:**
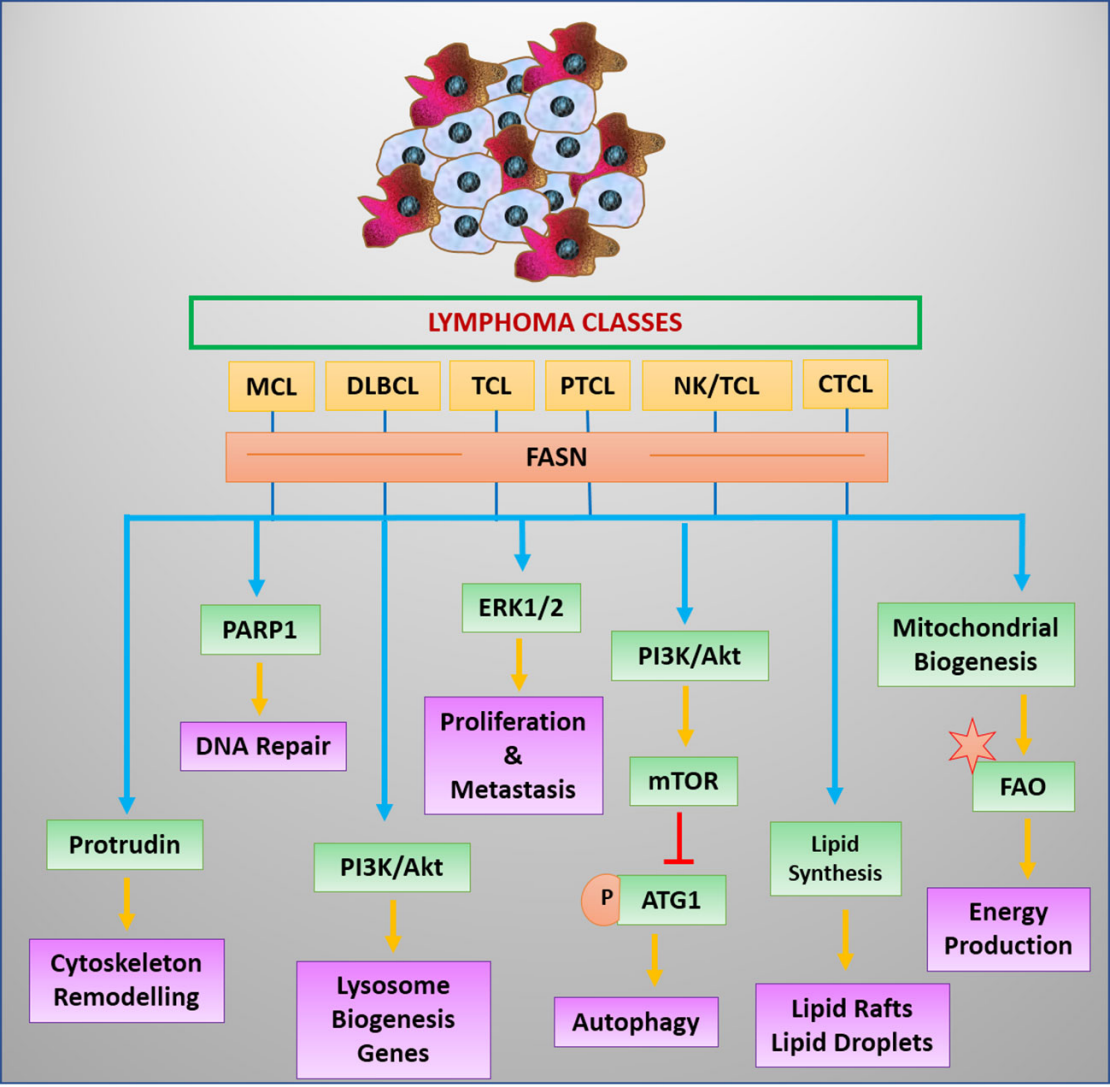
Regulators and benefits of lipogenesis in lymphoma/leukemia. Lipid synthesis and accumulation are regulated by survival signals in leukemia and lymphoma cells. Elevated lipid levels have advantages for cancer cells.

T-cell malignancies have been reported to modulate angiogenesis through high IL-17 expression (67). Moreover, IL-17 can mediate the expression of FABP, which coordinates with CD36 in the uptake of FA (113). IL-17 triggers the STAT3 signaling for transcriptional upregulation of FABP (113, 114). STAT3 favors the expression of CD36 in lymphoma cells (114). Nevertheless, mutational activation of STAT3 is a frequent genotype in malignant cells of T-cell origin (66). The enhanced expression or activities of regulators of transporters involved in fat uptake strongly indicates a high potential for lipid uptake in T-cell cancer. However, this notion warrants experimental validation (**Figure 4**).

## T-cell cancer and lipid droplets

T-cell malignancies are also observed to have a significant amount of lipid droplets (115). The quantitative and qualitative nature of lipid droplets depends on the form of cells as well as the exogenous source of lipids (18). Leukemic cells show an elevated level of lipid droplets when they were cultured in a medium containing an excessive amount of fatty acid or expose to a fat-rich diet (18, 116). Interestingly, leukemic cells were reported to force neighboring fat cells to provide precursors (free fatty acid) for their lipid droplet biosynthesis (117). Regulation of mediators of lipid droplet formation also plays an important role in the survival of cancer cells (118). KLF2 is one such negative regulator of FABP (119). In leukemia T cells, KLF2 is found to weaken the survival of cancer cells (120). KLF2 affects the proliferative ability of cancer cells by modulating the FABP5/PPARγ axis (118). Moreover, the therapeutic intervention targeting survival pathways of T-cell acute lymphoblastic leukemia also affects lipid droplet frequency (121). Collectively, these hints indicate the intriguing role of lipid droplets in the physiology of cancer cells in T-cell malignancies.

## Fatty acid oxidation in T-cell cancer

Fatty acid oxidation is shown to support the survival in stress conditions for tumor cells including those of blood cell origin (23, 30). The alteration in fat metabolism and dynamic balance between the synthesis and oxidation of fat molecules during dynamic settings is crucial for functionally differentiating as well as transforming malignant cells (30, 122). Wong et al. (2017) have reviewed the linkage of FAO with cancer formation in lymphocytes (123). In lymphoma cells, rewired fat metabolism was confirmed indispensable through targeted inhibition experiments. The inhibition of CPT1a caused cell growth arrest and induction of apoptosis along with mitochondrial damage in leukemia cells (70). Moreover, the leukemia cells were chemosensitized by a CPT1a inhibitor (124). Nevertheless, another FAO enzyme hydroxyacyl-CoA dehydrogenase/3-ketoacyl-CoA thiolase/enoyl-CoA hydratase (HADH) is a prognostic marker in hematological malignancies (125). Abnormal expression of HADH correlates with oncogenic transformation in various cell types including lymphoma (126). Targeting of HADH enzymes causes the arrest of cells in the G0 phase and chemo-sensitization toward doxorubicin (122). FAO breaks the stored fat and provides small carbon molecules to sustain the metabolic pathways in nutrient-starved conditions along with small lipid signaling molecules favoring aggressive cellular phenotype (77, 125). FAO is also suggested to provide acetyl CoA for the acetylation. One of the acetylation regulators is SIRT1 (87). Expression of SIRT1 in leukemia cell lines correlates with the degree of FAO (127). SIRT1 mediates the restructuring of lipid metabolism and elevates FAO levels in chemoresistant leukemia cells (127). Moreover, SIRT1 drives lymphomagenesis and maintains leukemia stem cell potential by modulating lipid metabolism (128). Induction of cell death after inhibition of SIRT1 (129) suggests the obligatory role of upholding FAO in T-cell leukemia cells. In chemoresistant leukemic cells, the necessity of FAO surpasses the requirement of cancer stem cells (130). The obligatory requirement of FAO in transformation, aggressive phenotype evolution, drug resistance, and functional regulation of enzymes along with fueling the synthesis precursors indicates its central role in T-cell malignancies.

## Concerted role of lipid metabolism in T-cell malignancy

The modulations of lipid metabolism in malignancies of T cells have an extensive impact on cellular conduct (13, 25, 70). The lipid metabolic rewiring, under the control of transcription factors and other regulators, affects the survival signaling and induction of death through the altered composition of lipids in T-cell cancer (23). Moreover, the structural impact due to the spatial abundance of specific lipid moieties implies the manifestation of chemoresistance (26, 130). The modulation of lipid metabolic wiring also affects their susceptibility toward antitumor immune response and potential to cause immunosuppression. Benefits of revamped lipid metabolism to T-cell cancer act through various dimensions. These means include integration of the regulators, their spatial arrangement, modulated expression profile, and impacting vulnerability to the action of death-inducing stimulants.

## Transcriptional regulation of metabolic players

Malignant cells of T lymphocyte origin also display a strenuous control of molecular troupes engaged in transcriptional regulation of lipid metabolism (13, 105). SREBPs are known as master regulators of lipid metabolism in normal cells as well as cancer cells. T-cell cancer has been reported to modulate cell physiology through an elevated level of SREBPs (31). The level of SREBP expression correlates with mTOR in a variety of leukemic cancer cells (31). SREBP favors the elevated expression of FASN in T-cell lymphoma (131). The transcriptional expression of FADS2 is also upregulated in malignant cells by SREBP (69). The expression of FADS2 is sought to be the provider of sapienate from palmitate (132). A higher level of sapienate provides the plasticity and makes cancer cells able to dodge the therapeutic inhibition of other key enzymes in lipid metabolism, such as SCD (69, 133). The “master transcriptional factors” also interplay with SREBPs and promote tumor progression through the modulation of lipid metabolism (134). The well-established regulator of cancer cell physiology, HIF-1α, is also linked with lipid metabolism (135, 136). The HIF-1α stabilization encourages fatty acid uptake and their accumulation in lipid droplets (135). T-cell malignancies also have heightened expression levels of HIF-1α (131, 137). HIF-1α induces the level of FABP and adipophilin (135). Adipophilin, a type of perilipin, is essentially required to initiate the formation of lipid droplets (17). The enhanced uptake driven by HIF-1α stabilization in cancer cells protects them from ROS-induced cell death (135). SREBP and HIF are linked with their activation through mTOR. The SREBP is also sought to compensate for the lipid requirement in the absence of HIF-1α stabilization through *de novo* synthesis of lipid moieties (135). HIF-1α-mediated proliferation utilizes a NOTCH1-mediated sequence of events in T-cell acute lymphoblastic leukemia (137). NOTCH1 also associates with lipid metabolism in T-ALL cells’ sensitivity toward therapeutic targeting (138). Interestingly, SREBP and HIF-1α are also essential for normal T cells to control the metabolic programming of effector T cells during the onset of the adaptive immune response (139). Lipid metabolism follows the inimitable course in the normal and different forms of cancer cells. The number of investigations on lipid metabolism in T-cell malignancies is still growing, and the broad spectrum of its regulation is being uncovered (13, 22).

## Lipid as a structural support

Lipid is the major constituent of biological membranes deciding the boundaries of cells and the organelles within. The varying composition of cellular lipid content as well as those of membrane influence cellular physiology and imply pathological consequences (3, 140). The rapidly dividing cells in malignant disorders need membranes as essential structural components (141). These varieties of lipid molecules have their structural advantages in providing physical strength and a distinctive assemblage of receptors and adhesion molecules (89, 141). The strength provides survival advantages to cancer cells to tolerate dynamic but otherwise hostile tumor microenvironments for other infiltrating cells (89). The assortment of receptors and unique membrane proteins in lipid rafts triggers the critical signaling events favoring cancer progression (68). This structural component of the membrane, lipid rafts, is targetable for the therapy of cancer (68, 141).

The structural assemblage of lipid rafts also has a critical role in maintaining cellular strength in T-cell leukemia (9). Structural disruption of lipid rafts in T-cell cancer cell lines leads to the induction of apoptosis through the depletion of survival signaling (9). These pieces of evidence indicate that lipid rafts are structurally holding the components of PI3K/AKT pathways. Structural compartmentalization through lipid rafts also affects the regulation of apoptotic cell deaths. The translocation of death receptors in leukemia cells by ether lipids is reported to mediate the induction of cell death (142). The approaches enforcing the localization of death receptors in lipid rafts have used lipid or lipid-derivative molecules (9, 142). This collectively indicates that the structural assemblage of lipid rafts is intrinsically programmed to prevent the recruitment and co-expression of components of cell death-inducing signals.

The structural integrity of lipid rafts is contributed by sphingolipids and cholesterols, regulated by SREBPs (29, 143). The cholesterol levels of cells as well as circulation correlate with the frequency of lipid rafts and caveolae in cancer cells (144). The contribution of cholesterol in strengthening the dynamic structures in the plasma membrane has been reported in T-cell cancer as well (131). The declined expression of SREBP along with increased membrane fragility of lymphoma of T cells indicates the role of cholesterol in the maintenance of physical strength. The altered lipid metabolism regulation not only affects tolerance to osmotic disturbances but also affects their resistance to the activity of anticancer drugs like cisplatin in T-cell lymphoma (105, 131). Cholesterol is known for conferring rigidity to the membrane of cells (145). The abundance of lipid rafts along with the level of cholesterol and other lipid moieties entopically retain survival and death regulatory protein molecules and adjust their function (9, 146). The retention of proteins and other signaling molecules in lipid rafts contributes to sustained and enhanced survival signals along with dynamic changes leading to the onset of metastasis (140, 144). The dynamic structural nature of lipid rafts serves as an essential determinant for migratory potential and invasive behavior of leukemia cells (140, 147).

Therapy resistance is also governed, at least partially, by the composition of lipids in the membrane of cancer cells (140, 141, 148). The membrane rich in cholesterol and with a low level of oxidizable fatty acids is observed in drug-resistant cancer cells (25, 148). The structural variation in the membrane due to altered lipid levels is also suggested to affect the susceptibility toward immune cell-mediated destruction of cancer cells (25). Depleting the cholesterol-synthesizing signaling protein has been shown to improve the immunotherapy response in cancers (149). The lower level of cholesterol improves the recognition of tumor cells by immune cells (25, 149). Elevated phospholipids, cholesterol, and sphingolipids in the membrane of cancer cells can blunt the antitumor immune response (25, 149).

## Lipid as a signaling moiety

Lipid-mediated signaling governs essential functions in transformed cells of T-cell origin (25). These bioactive lipid molecules involved in cellular signaling are commonly referred to as “signaling lipids” (24). The unique role of these signaling lipids is stated in tumorigenesis as well as in invasion and metastasis (8, 24, 150). Lipid-derived signaling molecules correlate with the outcome of the malignancies (8). Among many, broadly eicosanoids, phosphoinositides, and sphingolipids are major small signaling lipids that have a substantial role to play in cancer cell physiology (24). Interestingly, FABPs mediate signaling events along with their role in conveying fat molecules toward lipid droplets (24, 118). Moreover, FABPs regulate the epigenetic alteration in the DNA of leukemia cells (151). FABP5 is sought to supply ligands for PPARβ/δ and mediate the cancer progression (118).

Cholesterol has a differential impact on cellular signaling by assisting the lipid raft assemblage. Cholesterol in lipid rafts endorses receptor and receptor-complex aggregation (152). Lipid rafts mediate the signaling, through encompassed receptors, favoring the progression and evolution of malignant cells (9, 152). Moreover, the metabolic conversion of cholesterol generates oxysterol among many other bioactive compounds. Oxysterol is an activator of the liver X receptor, known to affect the function and metabolism in T cell-derived malignancies (153). Nevertheless, oxysterol-binding protein (OSBP)-related proteins (ORPs) favor the leukemogenesis of T cells in HTLV infection-induced carcinogenesis (154). Interestingly, OSBPs and OSBP-like proteins promote cancer cell survival through RAS signaling (155). Members of OSBPs are overexpressed in T-ALL cells (156). Although various reports indicate the tumor growth-promoting impact of oxysterols, it is worth mentioning that studies also demonstrated their tumor-inhibitory effects (157).

Cholesterol also serves as a precursor for steroid hormones including estrogen. Estrogen affects various human health-related conditions and is known to favor a variety of cancer types (158). Apart from conventional cancers of hormone-responsive tissues, lymphoma of T-cell origin also expresses receptors of gonadal steroidal hormones (159). Experimental pieces of evidence indicate that estrogen upkeeps the proliferation of murine T-cell lymphoma and reduces apoptotic cell death (159, 160).

## Lipid metabolism and prevention of cell death in T-cell malignancies

Metabolic acquaintances with various forms of cell dying including programmed cell death are long-established (73, 74, 133). The connections between lipid metabolism and their metabolites are also emerging as vital players in influencing cell death (161). Various lipid molecules, ceramide, and cardiolipin, the most common ones, are implicated in the regulation of mitochondria involving cell death in a Bcl2-regulated manner (162). Ceramide synthesis is elevated in T-ALL cells (163). Ceramide also confers protective effects against the induction of cell death by Bcl2 inhibitors (163). Interestingly, ceramide synthesis correlates with efficient TCR signal transduction and T-cell activation (164).

Another membrane lipid, cardiolipin, prevalent in the inner leaflet of the inner mitochondrial membrane flips to the outer leaflet during induction of apoptotic cell death after ROS detection (165). These transitions of cardiolipin occur even before changes known for assaying cell death including the detection of phosphatidylserine and DNA fragmentation (73, 74, 165). Upregulated levels of cardiolipins have been reported in types of cancer including leukemia (166, 167). Cardiolipin induction also favors the promotion of cancer through modulating cell death of T-cell cancer (166). Elevated cardiolipin aids in withstanding mitochondrial damage by interacting and facilitating the mitochondrial translocation of the BCR-ABL (167).

The elevated level of CPT1 and CPT2 observed in leukemia cells (168) correlates with the prevention of cell death (60). Elevated levels of CPT in leukemia cells were also found to be targetable through specific inhibitors (70). The pathways and intermediates of FAS also influence cell death (13, 87). The elevated level of FASN correlates with a low level of apoptosis in transplantable murine T-cell lymphoma (13). The inhibition of FASN was found to alter the expression profile of many apoptotic regulators such as Bcl2, p53, caspase, and HSP70 (13).

The high accumulation of cholesterol synthesis pathway intermediate has a protective effect against oxidative cell death in T-ALL cells (109). ACAT-1 triggers the esterification of cholesterol and promotes its storage or integration in the membrane. Cholesterol esterification correlates with the suppressed level of apoptosis in cancer cells (169). Various cancer cells including leukemia cells have an elevated level of ACAT-1 (170).

A concerted, linked, and independent role of lipid metabolites, enzymes, pathways, and their regulators can be suggested in the modulation of cell death. The CPT1 activity maneuvers the strength of FAO along with impacting apoptotic cell death (23). The studies indicate that specific inhibition of FASN induces apoptotic cell death without affecting CPT1 activity (13). These two contrasting pathways (FAS and FAO) operate exclusively; however, they can be suggested to avert cell death in distinct circumstances.

## Immunosuppression and immune escape

Metabolic links with tumor-induced immunosuppression are well established in T-cell malignancies (8). Stratified analysis correlates lipid metabolism-associated genes with the prognosis of leukemia (8). Moreover, immune response-related genes were found to be closely associated with lipid metabolism risk signature genes (8). These lipid metabolism risk signature genes largely affect the outcome of immunotherapy (8). A unique feature of the tumor microenvironment is the accumulation of specific lipids due to uniquely rewired lipid metabolism (27). Owing to the high glycolytic flux of tumor cells, the nutritional composition of the tumor microenvironment is largely governed by malignant cells (72). The fractional enrichment of long-chain fatty acid in the microenvironment impairs infiltrating cytotoxic T cells (27). Tumor cells drive the unavailability of preferred nutrients in the microenvironment for infiltrating immune cells. This leads to the uptake of low-density lipids, with their peroxidation causing diminished antitumor immune response (171). The cholesterol-rich tumor microenvironment has adverse consequences on antitumor immune response (153). Cholesterol metabolites also negatively affect the prevalence of cytotoxic T cells in the tumor microenvironment (143). The transcriptional regulation of 27-hydroxycholesterol is epigenetically governed by ZMYND8. Various studies have characterized the link of ZMYND8 with tumor growth promotion in leukemia cells (172) (11). The link between tumor-induced suppression of cytotoxic T cells also outreaches to the activity of Treg cells through modulation of lipid metabolism (143). Moreover, suppression of cytotoxic T cells decreases IFN levels favoring tumor growth promotion by macrophages, also known as tumor-associated macrophages (173). Such maintenance of tumor-associated macrophages requires SREBP-1-governed lipid metabolism (72, 173). Impairment of macrophages’ antitumor activity and promotion of growth by tumor-associated macrophages are well established in the lymphoma of T-cell origin (174, 175). The inhibition of SREBP makes leukemia cells susceptible to anti-PD-1 immunotherapy (176). Moreover, inhibition of SREBP1 negatively affects the tumor growth-promoting ability of tumor-associated macrophages (173). Therefore, it can be concluded that SREBP is, if not major, at least one of the key contributors to immunosuppression observed in cancers of T cells.

The level of eicosanoid lipids, like prostaglandins, is altered in leukemic cells (177). Lipids of eicosanoids have immunomodulatory consequences (177). The prostaglandin E2 promotes the tumor progression of T-acute lymphoblastic leukemia by affecting the cAMP signaling pathway (178). Prostaglandins have contrasting roles in the immune response. Prostaglandin E2 prevents the activation of helper T cells by inhibiting the signal transducers of T-cell receptor signaling (179). Production of eicosanoids (prostaglandins and leukotrienes) is governed by cyclooxygenase (COX) enzymes. A variety of hematological malignancies of T cells have elevated levels of COX enzymes (180, 181) and are therapeutically targetable (181). The inhibition of COX enzymes in T-cell lymphoma decreases the level of lactate (181). Lactic acidosis of the tumor microenvironment is detrimental to the antitumor immune response of macrophages and T cells (182). The level of lactate has been linked with sustained lipid metabolism in cancer cells (183). The lactate level in the tumor microenvironment of T-cell lymphoma correlates with the activation of M2-type tumor-associated macrophages (175).

The activity of FASN in cancer cells has staid acquaintances with the regulation of antitumor immune response. The elevated level of FASN, observed in T-cell tumors, correlated with the decreased level of antitumor response by tumor-infiltrating macrophages (184). Moreover, inhibition of FASN through small molecule inhibitors withdraws the suppression of hematopoietic differentiation of bone marrow cells (184). This indicates the essentiality of FASN modulating antitumor immune response in hematological malignancies.

Lipid metabolism has a diversified impact on cancer physiology and cancer-immune cross talk. The varied arms of lipid metabolism, including lipid uptake and synthesis of fatty acids and sterols, along with lipid derivatives not only modulate the tumor cell survival potential but also affect their immune sensitivity and antitumor immune response. The lipid metabolites affect the hematological differentiation, tumor tissue infiltration, and subsequent antitumor activation of immune cells. Moreover, the intervened disposition of lipid metabolism regulates the level of other immunosuppressive metabolites, such as lactate, in the tumor microenvironment. Many of these pathways and metabolites associated with lipid metabolism are found altered in malignancies originating from T cells and modulate the immune response.

## Lipid metabolism and chemoresistance interface in T-cell cancer

Chemoresistance is a leading obstacle in the clinical management of malignant disorders (74). Malignancies originating from T cells are also no exemption from this (26, 185). Moreover, the onset of chemoresistance correlates with metabolic alterations in T-cell cancer (130, 185, 186). Although a majority of investigations aiming to link metabolism are mainly focused on glycolytic metabolism (187, 188), the role of altered lipid metabolism in the onset and maintenance of chemoresistance is gaining attention (130, 186). Hyperglycoytic phenotype has been linked with aggressive cancer cells (189). A major product of glycolytic metabolism, lactate affects both lipid metabolism and chemoresistance (74, 183). Oxidative metabolism of fat contributes to the maintenance of chemoresistance along with conserving the stem cell ness (186). Leukemia cells resistant to chemotherapy preferentially rely on their oxidative metabolism of fatty acids (130). Nevertheless, upregulated CD36 is required for these chemoresistance leukemic cells (130). CD36 mediates the IL-6-driven resistance against standard chemotherapeutic agents through elevated fatty acid uptake in leukemia cells (190). CD36 and autophagic events are also inversely regulated (191). Suppressed autophagy is linked with chemoresistance in T-ALL cells (192). CD36-mediated lipid accumulation provides ATP through FAO, which energizes the machinery responsible for manifesting resistance in leukemia cells (130).

Sphingolipids are also linked with the chemoresistance of cancer cells including T-ALL cells (20, 191). Augmented levels of ceramide synthase and its product ceramide have been confirmed to elevate therapy resistance in T-ALL cells (163, 173). Ceramide synthase provides ceramide, a precursor of sphingolipids. Ceramide level correlates with the expression of ABCB1 (193). Daunorubicin-resistant cells of T-cell leukemia display elevated levels of ABCB1 expression (194). Obstructing the assembly of Fas and Fas-associated protein with the death domain in T-cell leukemia by ceramide synthase is suggested as one of the mechanisms preventing the induction of cell death by therapeutic drugs (163).

Another enzyme of lipid metabolism, sphingosine kinase, also has an implementation in chemoresistance (20). Sphingosine kinase-1 confers resistance to standard chemotherapeutic drugs in cancer cells of hematopoietic origin (195) and has been suggested as a putative target for the therapy of lymphocytic leukemia (196). Moreover, sphingosine kinase contributes to tumor cell aggressiveness by aiding in the ceramide pathway in leukemia cells (197). Sphingosine kinase level correlates with the unfolded protein response in T-ALL cells (196). The unfolded protein response contributes to the rapid progression of the disease as well as aids in chemoresistance in leukemic cells (198). The regulatory connection of unfolded protein response ranges to other arms of lipid metabolism such as cholesterol and fatty acid synthesis and oxidation (199). Cholesterol can induce unfolded protein response leading to phenotypic alterations (200). An enzyme hydroxy-3-methylglutaryl-CoA synthase 1 (HMGCS1), involved in cholesterol synthesis, modulates the unfolded protein response in leukemia cells (201). Nevertheless, high cholesterol levels are also connected to resistance to chemotherapy (202). Elevation in cholesterol level, through either sequestration or biosynthesis, is suggested as a means to protect leukemia cells from chemotherapeutic drug-induced cell death (203). Dong et al. (2010) indicated the involvement of cholesterol biosynthesis with chemoresistance in T-ALL cells against doxorubicin using stable isotope labeling by amino acids in cell culture (SILAC) approach (204). Supplementation of cholesterol in culture media had a salvage effect on drug-induced death in Jurkat cells (204). Moreover, the level of membrane cholesterol regulates the activity of ABC protein and the manifestation of chemoresistance (205). The high level of cholesterol and sphingolipids in lipid rafts of T-ALL cells contributes to resistance against therapy (145). Interestingly, lipid rafts in lymphoma cells are demonstrated to retain the constitutively expressed apoptotic protease-activating factor-1 (APAF1) to prevent cytochrome c-mediated cell death in response to chemotherapeutic drugs. Lipid rafts also contribute to the activity of FASN in cancer cells (12). Inhibiting the FASN through RNA silencing as well as chemical inhibitor reverses the resistance of cancer cells against Herceptin (12). Pharmacological inhibition of FASN through orlistat modulated the tumor microenvironment and reversed the drug resistance in a murine T-cell lymphoma (105). FAO pathways are also suggested to affect the sensitivity of cancer cells toward the action of therapeutic drugs. In leukemia cells, the resistant populations have a high oxidative metabolic rate when compared with the susceptible population of cells (130). FAO is suggested to provide the required ATP to drive survival when other sources are blocked therapeutically (22).

The varied dimensions of lipid metabolism support the survival of malignant cells of T cells by playing their energy source, regulating the function of drug exporters, signaling cascade, and disrupting the assembly of death inducers. Their contribution to the onset of chemoresistance underlies activating the survival signals and providing nutrients, and energy and fueling the molecular pumps to expel the drug molecules. Targeting the constituents of lipid metabolism has chemosensitizing and therapeutic consequences on T-cell malignancies. This can be explored as an adjuvant strategy in the clinical management of T-cell malignancies.

## Lipid metabolism: a novel therapeutic target for T-cell malignancy

Therapeutic targeting of cancer metabolism has clinical relevance (206). The successful attempt of targeting metabolism in T-cell malignancies has been carried out in various investigations (29, 131, 207). Targeting lipid metabolism in cancers of T-cells has demonstrated promising results (13, 22, 105, 208). Moreover, modulation of lipid metabolism or their regulators is found to modulate an effective therapeutic intervention against T-cell malignancies (131). Targeting lipid metabolism has a direct cytotoxic effect on cancer cells as well as an adjuvant effect through potentiating the effect of chemotherapeutic drugs (130, 208). Nevertheless, the consequences of targeting lipids include improved immunotherapy-mediated destruction of leukemic cells (175, 208).

In a murine transplantable T-cell lymphoma, pharmacological inhibition of FASN has a direct inhibitory effect on the survival of tumor cells (13). The augmented level of the pro-apoptotic molecule such as p53, and caspase and diminished level of anti-apoptotic Bcl2, HSP70 were observed in lymphoma cells of T-cell origin exposed to orlistat, a FASN inhibitor (13). FASN inhibition also has a chemosensitizing effect in T-cell cancer. Modulated tumor microenvironment in response to FASN inhibition along with the reversal of multidrug resistance phenotype was linked with decreased expression of ABC proteins (105). Augmented differentiation and antitumor activation of macrophages indicate decreased immunosuppression in the tumor-bearing host of a T-cell lymphoma treated with a FASN inhibitor (184). Downregulated expression of PD-L1 on human leukemic T cells by orlistat can be linked with their declined immunosuppressive ability after FASN inhibition (15). Interfering FASN expression by either RNAi or EGCG alleviates the effectiveness of differentiation therapy in leukemia (209). Various other plant-derived chemicals have the potential to inhibit leukemic cell lines (210). Phytochemicals can suppress FASN leukemic cells. Ginger extract, gallic acid, cerulenin, and ginkgolic acids have demonstrated promising success in inhibiting leukemia cells by diminishing the FASN activity (104, 211, 212).

Many investigations suggest CPT1A as a therapeutic target in malignant disorders (70, 168, 213). CPT1A is responsible for fueling FAO through translocating cytoplasmic fatty acid to mitochondria (70, 213, 214), thus aiding in the dynamic and robust energy requirement of malignant cells (213). One CPT1A inhibitor, (*R*)-*N*-(tetradecyl carbamoyl)-amino carnitine (ST1326), inhibits the occurrence of lymphoma (28) and leukemia (70). The inhibition of CPT1A and associated FAO in leukemia cells by etomoxir or ranolazine augmented the efficacy of ABT-737-mediated cell death through pro-apoptotic Bak protein (124). The inhibition of leukemic T cells by *N*-farnesyl-norcantharimide (NC15) correlates with alteration in fatty acid metabolism genes (215). Moreover, resistant T-ALL cells show an altered rate and relatively high dependence on FAO over exogenous glutamine (71). This suggests the essential role of FAO for hematological malignancies during drug-induced stress conditions.

Cholesterol metabolism is also one of the effective targets in the treatment of T-cell malignancies (29). The metabolism and uptake of cholesterol in child T-ALL have marked differences from normal cells. Statins are one of the common cholesterol-lowering agents through the inhibition of HMGCR. Simvastatin, atorvastatin, fluvastatin, and lovastatin have the potential to inhibit leukemia cells in both laboratory experiments and clinical settings (216–218). Moreover, statins are adjuvant to other drugs and chemosensitizes leukemia cells (217). Lowering cholesterol levels improves the chemosensitivity of leukemia cells to the activity of rituximab and fludarabine (202).

Anticancer activities of cholesterol-lowering statins are also affected by the activity of SREBPs (219, 220). The weak statin sensitivities are linked with the activity of SREBPs and HMGCR (219, 221). This notion points out the targetable nature of these two regulators of lipid metabolism. The chemical targeting of SREBP or SCAP/SREBP complex in acute lymphoblastic leukemia showed promising success (220, 222). SREBP inhibition in cutaneous T-cell lymphoma impairs the survival of malignant cells (31). However, malignant cells tend to compensate for SREBP inhibition by escalating the FASN activity. Therefore, dual inhibition of SREBP and FASN has enhanced suppressing effect on survival of T-cell cancer (31). SREBP mediates the MYC-induced tumorigenesis in hematopoietic malignancies (223) and co-regulates the FASN expression (223, 224). Various natural compounds are suggested to inhibit SREBP expression in tumor cells. One of the plant compounds, methyl jasmonate, decreases the levels of SREBP as well as FASN expression in cells of T-cell lymphoma (131). The declined expression of SREBP in T-cell lymphoma treated with methyl jasmonate correlated with decreased survival as well as augmented susceptibility to the activity of cisplatin (131). Cholesterol and sphingolipids contribute to the integrity and functioning of lipid rafts (29, 143). Lipid raft integrity is essentially required for the invasive behavior of T-cell leukemia (140). The destruction of lipid rafts leads to the disengagement of transient receptor potential vanilloid, type 6 (TRPV6) calcium channel, which is suggested as the underlying mechanism (140).

Lipid metabolism has several molecular targets for therapeutic interventions of T-cell cancer. They variedly range from molecules involved in lipogenesis as well as in the oxidation of lipids. Lipid metabolism inhibition in tumor cells has direct cytotoxic consequences on T-cell malignancies. In many cases, targeting one arm of lipid metabolism is being compensated by the other arm. However, the exclusive role of each component in different stages of tumor initiation, progression phenotypic evolution as well as invasion, is indispensable by malignant T cells. Targeting lipid metabolism also offers the reversal of therapy resistance. Considering the promising results of targeting lipid metabolism indicated by available laboratory or primary clinical investigation, further investigation is needed to strengthen its clinical relevance and applicability.

## Conclusion

The distinctive metabolic setup for lipids governs the numerous aspects of the physiology of T cells as well as malignancies derived from T cells (184). During different stages of maturation and differentiation, lipid metabolism acts as a maneuvering tool in the life of T cells (33). The differential lipid metabolism contributes to the necessity during different stages of T-cell life, and its divergent operations also support diverse requirements of T-cell subsets (32). Moreover, regulatory events controlling T-cell response are also backed by unique lipid metabolism in regulatory cells (10, 54). Nevertheless, divergent lipid metabolism also contributes to the initiation and maintenance of T-cell malignancies. The anabolic and catabolic arms of lipid metabolism [biosynthesis, accumulation, and oxidation of lipid moieties] are inter-regulated. In T-cell malignancies, FASN-mediated FA synthesis provides essential lipid constituents to meet the demand for biological building blocks to sustain rapid cell division (13). The lipid molecules also provide essential survival advantages by strengthening the structure of cells by modulating the composition of the membrane. Moreover, the specific composition of lipids in cell membranes contributes to maintaining the lipid rafts in T-cell cancer (9, 68). The integrity of lipid rafts essentially maintains the surface expression of receptor and adhesion molecules involved in enhanced cell survival signaling and membrane dynamics during invasion and metastasis (9, 25, 68). Nevertheless, the specific lipid composition of cells is also linked with immunosuppression and immunoescape measures in malignancies of T cells (8, 13). The elevated rigidity provided by high cholesterol levels along with enriched non-oxidizable lipid content in the membrane confers resistance to chemotherapeutic drugs (47, 200). Apart from structural advantage, lipid provides reserve fuels for dynamic and robust requirement during metastatic and chemoresistance manifestation (3, 17).

Rewiring of lipid metabolism generates molecular moieties involved in cellular signaling and regulates cancer progression in hematological malignancies. The signaling lipids maintain the required level of survival signaling and prevent the induction of cell death in T-cell cancer (12, 163). Various cancer types including T-cell cancer have unique transcriptional regulations affecting the cell physiology and favor the progression of malignancies (23, 31).

The various arms in lipid metabolism act in a concerted form in T-cell malignancies. The accumulation of lipid molecules is also contributed by the uptake of free lipid molecules from the cellular exterior, their incorporation, and storage in the form of lipid droplets. The qualitative and quantitative abundance of lipid type and forms affects cellular stress including endoplasmic stress and unfolded protein response leukemic cells (16, 17, 147, 198). The oxidation of reserve lipids offers energy to perform various cellular functions as well as to provide precursors for numerous bioactive lipids (17, 23, 145).

Collectively, reprogrammed lipid metabolism aids in phenotypic characteristics of T-cell malignancies in a much-intervened fashion. The interknitting of lipid metabolism is limited to their catabolism and anabolism, but they also connect their dots with carbohydrate and amino acid metabolism. The indispensability of lipid metabolism has been suggested to be a promising therapeutic target. Targeting FA biosynthesis and FA oxidation has been attempted in preclinical and clinical investigations, and both have encouraging outcomes against malignancies of T cells (13, 23, 104, 209). The direct inhibition of cancer progression by targeting lipid metabolism as well as alleviation of chemo- and immunotherapy response has been demonstrated by various investigations (104, 129, 209, 217). Considering the demonstrated as well as envisaged impact of lipid metabolism in T-cell malignancies, investigations exploring the connections between hallmark characters with the unambiguous arrangement in lipid metabolism are further warranted.

## Author contributions

NV, DS, AK and SS conceptualized the study. AM, YR, VS and NV contributed to the literature search and analysis. All authors contributed to the writing of the manuscript. Critical evaluations and revisions were made by NV, DS, AK and SS. All authors contributed to the article and approved the submitted version.
